# Impact of Different Ratios of Lignin Waste and Liquid Glass on the Performance Characteristics of Biopolyurethane Foams

**DOI:** 10.3390/polym15040818

**Published:** 2023-02-06

**Authors:** Agnė Kairytė, Jurga Šeputytė-Jucikė, Sylwia Członka, Sigitas Vėjelis, Saulius Vaitkus

**Affiliations:** 1 Laboratory of Thermal Insulating Materials and Acoustics, Institute of Building Materials, Faculty of Civil Engineering, Vilnius Gediminas Technical University, Linkmenų st. 28, 08217 Vilnius, Lithuania; 2 Institute of Polymer & Dye Technology, Lodz University of Technology, 90-924 Lodz, Poland

**Keywords:** lignin waste, liquid glass, biopolyurethane foam, performance characteristics, filler and polymer interaction

## Abstract

In the current study, biopolyurethane foam was modified with 2.5–10 wt.% lignin waste (LigW) and liquid glass (LG)-modified LigW particles at different LigW/LG ratios—1:1 and 1:2—and their impact on performance characteristics—i.e., rheology, foaming times, apparent density, thermal conductivity before and after aging, dimensional stability at ambient and elevated conditions, compressive and tensile strengths, short-term water absorption by partial immersion, and water vapor permeability—was determined and evaluated. Structural analysis was implemented and structural parameters were taken into consideration as well. During the study, it was determined that 2.5–10 wt.% particles at the LigW/LG ratio of 1:2 showed a superior impact on the physical and mechanical properties of bioPUR foams. The apparent density only insignificantly increased and was in a density range suitable for commercially available polyurethanes. For particles at 10 wt.% and LigW/LG ratio of 1:1, the thermal conductivity value improved by 3.2%, the compressive strength increased by 153%, while the tensile strength improved by 23.5%, indicating sufficient interfacial adhesion between the filler and polymer matrix. Moreover, the short-term water absorption by partial immersion remained almost unchanged, while the water vapour diffusion resistance factor improved from 43 to 48. Additionally, the incorporation of LigW/LG 1:1 and LigW/LG 1:2 particles made it possible to obtain dimensionally and structurally stable closed-cell bioPUR foams for possible application as thermal insulation in building envelopes.

## 1. Introduction

Polyurethane is among the most promising polymers on the market, with the widest potential application. It can be successfully applied in the form of flexible, semi-rigid, and rigid foams, coatings, adhesives, and elastomers. Rigid polyurethane foams are commonly used in the construction sector as a component with superior heat insulation and mechanical properties [1]. Currently, the production of rigid polyurethane foams with sufficient or high mechanical performance, thermal insulation and long-term stability are mainly dependent on petroleum-based polyols [2,3]. However, new trends associated with sustainable development are encouraging the replacement of petroleum-based polyols with renewable hydroxyl derivatives synthesized from natural or used oils [4,5]. Another sustainable focus in the production of polyurethanes is their modification with recycled components, such as eggshell biowaste, waste tire rubber, etc. [6,7]. Such an approach increases the possibility of reducing the price of polyurethane composites, improving their performance and inducing waste utilisation [8]. Additionally, the utilization of carbon-neutral resources may help alleviate the global warming crisis, decrease the dependence on fossil-based resources, and obtain novel products based on renewable resources.

Lignocellulose is a promising renewable carbon resource for the production of bio-based materials. It is mainly composed of the following biopolymers: cellulose, hemicellulose, and lignin, with small amounts of ash and other extractives. Cellulose and hemicellulose have been extensively implemented in biogas, bioethanol, pulp engineering, and other biorefinery industries [9]. According to study [10], lignin production reaches over 50-million tons annually, and it is obtained as a side product in the paper industry, so the amount produced is expected to increase in the future. However, due to its complex structure, lignin utilization has always been an obstacle in the biorefinery industry, which is the reason why approximately 95% of lignin is instead burned to supply energy [11]. A high-value utilization of lignin waste is of great importance and the application of lignin for the synthesis of polyurethane foams can be one such utilization solution.

Most of the studies focus on the combination of lignin as a macromonomer in polyurethane synthesis, and three main approaches have been developed [12]: (1) direct usage of lignin without any treatments, when the lignin can be directly reacted with isocyanates with or without polyols; (2) utilization of lignin after solvent fractionation; and (3) utilization of lignin after chemical modification. Gondaliya and Nejad [13] reported partial substitution of polyol with 20 wt.% lignin in the synthesis of flexible polyurethane foams and concluded that the most important parameter determining sufficient performance of such foams is the hydroxyl content in the lignin. Luo et al. [14] and Li et al. [15] showed that the incorporation of lignin into polyurethane foam resulted in a non-homogeneous cellular structure with increased open porosity. Additionally, Li and Ragauskas [16] reported that the incorporation of lignin as a filler into polyurethane foam caused dimensional changes and reduced its mechanical performance. To overcome these shortcomings, novel approaches should be considered for the successful usage of lignin in polyurethane foams. To the authors’ knowledge, the current study is the first one to discuss liquid glass spray-coating for the more successful application of lignin waste in bio-based polyurethane (bioPUR) foams.

The aim of this study is to determine whether additional modification of the lignin waste (LigW) surface could improve its performance and the reaction profile of bioPUR foams. The suggested technology includes spray-coating LigW particles with liquid glass (LG) at different LigW/LG ratios. Moreover, the unmodified LigW and LG-modified LigW particles were further incorporated into rigid bioPUR foams and their performance characteristics were determined in terms of apparent density, thermal conductivity before and after aging, dimensional stability at ambient and elevated conditions, compressive and tensile strengths, short-term water absorption, and water vapour permeability.

## 2. Materials and Methods

### 2.1. Materials

Two bio-based polyols were incorporated to produce bioPUR foams—polyol from rapeseed oil BioPolyol RD (SIA PolyLabs, Riga, Latvia) having a hydroxyl value of 350 KOH/g, and polyol from sucrose PETOL 400-4G (Oltchim, Râmnicu Vâlcea, Romania) having a hydroxyl value of 421 KOH/g. As isocyanate, polymeric 4,4-diphenylmethane diisocyanate Lupranat M20S (NCO = 31.5%) was selected (BASF, Ludwigshafen, Germany). Distilled water was used as a blowing agent. Polycat 9 (Air Products and Chemicals, Inc., Allentown, PA, USA) was incorporated as a blowing and gelling catalyst. ST-52 (Shijiazhuang Chuanghong Technology Co., Ltd., Shijiazhuang, China) was used as a surfactant for the purpose of obtaining a regular cellular structure. LigW was incorporated as bioPUR foam filler, which has an initial moisture content of 1 wt.%. It was supplied by a local company (JSC Lignineko, Kėdainiai, Lithuania). LG is a sodium silicate which was purchased from JSC Lerochemas, Klaipeda, Lithuania.

### 2.2. Preparation of BioPUR Foams

First, the LigW was sieved in order to eliminate contamination with rocks. Then the LigW particles were sprayed with LG at ratios of 1:1 and 1:2, thoroughly mixed together at 1200 rpm for 5 min, and dried at 105 °C for 24 h. The obtained LG-coated LigW particles were cooled down to (23 ± 5) °C temperature and stored in sealed containers. The prepared LigW/LG 1:1 and LigW/LG particles had a moisture content of 0.4 wt.% and 0.3 wt.%, respectively.

Before adding the LigW and LigW/LG particles, a polyol premix was prepared. Firstly, scaled amounts (Table 1) of polyols, distilled water, catalyst, and surfactant were thoroughly mixed. Secondly, the polyol premix obtained was divided into four parts—control polyol premix, polyol premix for LigW particles’ addition, polyol premix for LigW/LG 1:1 particles’ addition, and polyol premix for LigW/LG 1:2 particles’ addition. Thirdly, the prepared polyol premixes were mixed with the respective amounts of LigW, LigW/LG 1:1, and LigW/LG 1:2 particles. Finally, the four prepared polyol premixes were thoroughly mixed with isocyanate for 10 s at 1800 rpm. The prepared foaming mixtures were poured into open moulds and left to cure at (23 ± 2) °C and (50 ± 5)% relative air humidity conditions.

### 2.3. Test Methods

The moisture content of the particles was determined for three samples using a moisture analyser KERN DAB (KERN & Sohn GmbH, Balingen, Germany).

The granulometry of the particles was determined using a set of sieves consisting of bottom, 0.1 mm, 0.25 mm, 0.5 mm, 1 mm, 1.25 mm, and 2.5 mm-sized sieves.

Images of the polyol premixes with various amounts of LigW, LigW/LG 1:1, and LigW/LG 1:2 were captured using an optical microscope Smart 5MP PRO (Delta Optical, Gdańsk, Poland) capable of magnification up to 300 times.

The dynamic viscosity of the polyol premixes with the respective amounts of particles was determined by an NDJ-8S digital rotational viscometer (Wincom Company Ltd., Changsha, China) having a rotor velocity of 0.3–60 rpm and measurement range of 10–2,000,000 mPa s.

The characteristic reaction profile—cream, gel, and tack-free times—was determined according to EN 14315-1 [17], Annex E requirements. All components were stored at (20 ± 1) °C before the measurement. The foaming times were measured using an electronic stopwatch with an accuracy of 0.5 s.

The structural studies of the LigW particles, LigW/LG 1:1 particles, LigW/LG 1:2 particles, control bioPUR foam, and bioPUR foam with the respective particles were conducted using a scanning electron microscope (SEM) Helios NanoLab 650 (Oxford Instruments, Abingdon, UK). The images obtained were further analysed for the average cell size of the bioPUR foams using the ImageJ software. The elemental composition of the LigW, LigW/LG 1:1 and LigW/LG 1:2 particles was determined with an X-ray spectrometer INCAEnergy (Oxford Instruments, Abingdon, UK) with an X-max detector.

The linear dimensions of the bioPUR foam samples were measured in accordance with EN 12085 [18], the apparent density—EN 1602 [19]—for samples having the size of 50 mm × 50 mm × 50 mm.

Determination of the closed-cell content was carried out according to EN ISO 4590 [20] method 2 for 100 mm × 30 mm × 30 mm sized samples.

Initial thermal conductivity and thermal conductivity after ageing measurements were conducted at an average testing temperature of 10 °C on 300 mm × 300 mm × 50 mm sized samples based on EN 12667 [21] requirements using a heat flow meter FOX 304 (TA Instruments, Newcastle, DE, USA) with an active edge insulation. The direction of the heat flow was upwards during the test. The difference between cold and hot plates was 20 °C. The ageing procedure for samples was carried out according to EN 14315-1 [17], Annex C.

Initial dimensional changes at normal laboratory conditions (23 ± 5) °C and (50 ± 5)% were evaluated 30 min and 1 day after production by calculating relative changes in the length, width, and thickness according to Equation (1):

(1) $$\Delta \varepsilon =\frac{b_{1}-b_{2}}{b_{1}}\cdot 100$$

where Δ*ε* is the initial dimensional change in the length, width, or thickness, %; *b*_1_ is the length, width, or thickness of the sample 30 min after production, mm; *b*_2_ is the length, width, or thickness of the sample 1 day after production, mm.

Dimensional stability under elevated conditions was carried out in accordance with EN 1604 [22]. Samples were maintained at (70 ± 2) °C and (90 ± 5)% relative humidity for 48 h in a climatic chamber Feutron 3522/51.

The short-term water absorption of the control bioPUR foam and bioPUR foams with LigW, LigW/LG 1:1, and LigW/LG 1:2 particles was carried out according to ISO 29767 [23] for samples having the size of 200 mm × 200 mm × 50 mm. After the test, samples were drained of excess water for 10 min using a drainage stand.

A water vapour permeability test was carried out for samples having a size of 100 mm × 100 mm × 50 mm based on EN 12086 [24] requirements. Plastic plate assemblies with samples and salt were maintained at (23 ± 1) °C and (50 ± 3)% moisture. The required moisture of (93 ± 3)% in the assemblies was achieved with potassium nitrate solution.

The compressive and tensile strengths were determined in accordance with EN 826 [25] and EN 1607 [26], respectively, for the samples having the size of 50 mm × 50 mm × 50 mm using a testing machine H10KS Hounsfield (Tinius Olsen Ltd., Surrey, UK).

## 3. Results and Discussion

### 3.1. Characterisation of LigW and LigW/LG Particles

**Figure 1:**
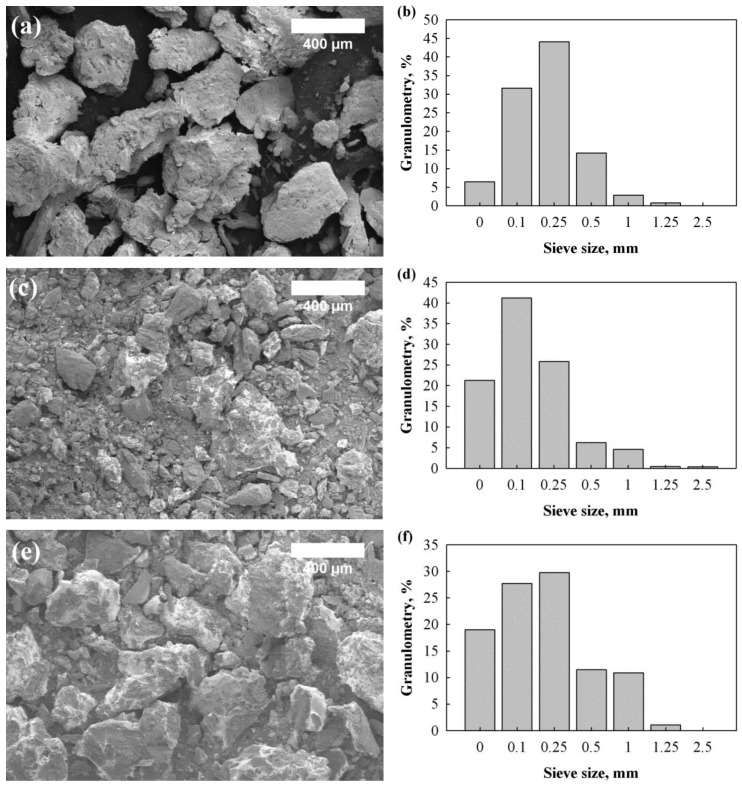
SEM images and particle size distribution of LigW and LigW/LG particles: (**a**) LigW particles (magnification ×100); (**b**) granulometry of LigW particles; (**c**) LigW/LG 1:1 particles (magnification ×100); (**d**) granulometry of LigW/LG 1:1 particles; (**e**) LigW/LG 1:2 particles (magnification ×100); (**f**) granulometry of LigW/LG 1:2 particles.

The external structure of the LigW and LigW/LG particles was analysed from the SEM images presented in Figure 1. It was observed that, before modification, the surface of the LigW particles was rougher and the edges of each particle were sharper (Figure 1a). Additionally, the LigW particles contained lignocellulose biomass, which was not eliminated from the current study and used as is. The overall structure of the LigW/LG 1:1 (Figure 1c) and Lig/W 1:2 (Figure 1e) particles became smoother with the reduced sharpness of the edges. It was also revealed that the surface of all the LG-modified particles was uniformly coated. However, Miedzińska et al. [27] indicated that surface modification with such materials as casein, chitosan, and potato protein can lead to the formation of a rough surface, which is suitable for better interfacial adhesion between the particles and polymer matrix.

The particle size distribution of the unmodified lignin waste (LigW), modified lignin waste with the LG ratio of 1:1 (LigW/LG 1:1), and modified lignin waste with the LG ratio of 1:2 (LigW/LG 1:2) is presented in Figure 1. The results show that the size of the modified and unmodified lignin waste was up to 2.5 mm. The highest percentage of particles was observed at 0.25 mm for LigW, 0.1 mm for LigW/LG 1:1, and 0.25 mm for LigW/LG 1:2 fillers. The smaller particles in LigW/LG 1:1 were obtained due to additional milling after the sprayed coating was hardened, as the particles were partly agglomerated, while the larger particles in LigW/LG 1:2 were fully agglomerated and were highly resistant to additional milling. In order to check the coating efficacy for LigW with LG, the elemental composition of each of the particle types was determined and the results obtained are presented in Table 2.

It can be clearly seen that the percentage of the main elements of LG such as Si and Na increases with the increase in the LG ratio from 1:1 to 1:2, thus confirming the successful coating of the lignin waste particles. The additional elements detected, such as sodium, aluminium, silica, sulphur, calcium, and iron, come from impurities, as the LigW was obtained from paper and pulp industry landfill [28].

### 3.2. Characterisation of Polyol Premixes and Foaming Mixtures

**Figure 2:**
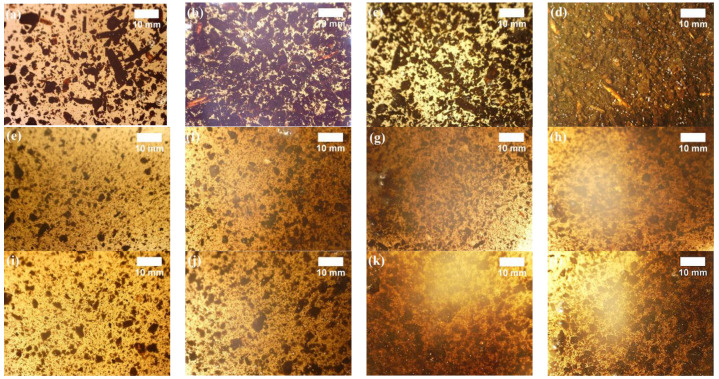
Particle distribution in polyol premix: (**a**) 2.5 wt.% LigW; (**b**) 5 wt.% LigW; (**c**) 7.5 wt.% LigW; (**d**) 10 wt.% LigW; (**e**) 2.5 wt.% LigW/LG 1:1; (**f**) 5 wt.% LigW/LG 1:1; (**g**) 7.5 wt.% LigW/LG 1:1; (**h**) 10 wt.% LigW/LG 1:1; (**i**) 2.5 wt.% LigW/LG 1:2; (**j**) 5 wt.% LigW/LG 1:2; (**k**) 7.5 wt.% LigW/LG 1:2; (**l**) 10 wt.% LigW/LG 1:2.

The rheological behaviour of polyol premixes is one of the most important parameters for sprayed or board-like PUR because the rheology gives information about the dynamic viscosity of the fluids. Moreover, the particle size is the key parameter that describes the filler and its behaviour in the polyol dispersion [29], which describes the possibility of using fillers in PUR systems. Optical imaging was employed to present the behaviour of LigW (Figure 2a–d), LigW/LG 1:1 (Figure 2e–h) and LigW/LG 1:2 (Figure 2i–l) particles in polyol premixes, while Table 3 presents the dynamic viscosity and peak temperature results of the particle-filled polyol premixes.

It can be clearly seen from Figure 2a–d that the LigW particles and lignocellulose biomass are highly accumulated, some of them agglomerated in the polyol premix. The lighter parts in the optical images indicate the polyol premix, although it cannot be detected at 10 wt.% LigW (Figure 2d) as the dynamic viscosity of that mixture increases by almost 700% (Table 3). On the contrary, the incorporation of LigW/LG 1:1 particles resulted in polyol premixes with scattered particles (Figure 2e–h) having a dynamic viscosity reduced by 70% when comparing the polyol premixes with 10 wt.% LigW and LigW/LG 1:1 particles. However, the incorporation of LigW/LG 1:2 particles resulted in a denser distribution in the polyol premixes (Figure 2i–l), as the average size of the LigW/LG 1:2 particles increased after modification with the LG ratio of 1:2. The dynamic viscosity of such premixes increased by 1% when comparing 10 wt.% LigW/LG 1:1 and LigW/LG 1:2, but reduced by 70% when comparing 10 wt.% LigW and LigW/LG 1:2. The difference in the increase in the dynamic viscosity of the polyol premixes with LigW and LG-modified LigW particles may be due to the plasticising effect of the LG-modified LigW particles, which reduces the visible agglomeration of the fillers used. Similar tendencies in the dynamic viscosity increase of particles as filler in PUR foams were shown in the study by Leszczyńska et al. [30], where polyurethane foams were reinforced with ground chokeberry pomace, raspberry seeds, walnut shells, and hazelnut shells. The authors also concluded that the change in dynamic viscosity can be explained by the greater difference in the size of the filler particles and the larger amount of irregular and rough particles.

Table 3 presents the influence of the LigW, LigW/LG 1:1, and LigW/LG 1:2 content on the foaming kinetics of the prepared bioPUR foams. Interestingly, the LigW particles increased the cream time of the polyol mixtures. This effect is commonly observed for PUR and bioPUR composites [31,32,33].

However, the improvement in the parameter can be seen for bioPUR foam with LigW/LG 1:1 and LigW 1:2 particles. For instance, even though the dynamic viscosity increases, the introduction of LigW/LG 1:1 particles reduced the cream time from 28 s to 25 s while LigW/LG 1:2 particles reduced the cream time from 30 s to 18 s at 2.5 wt.% and 10 wt.%, respectively. This effect can be attributed to the fact that the LG sufficiently coats the surface of the particles, thus “locking” the free hydroxyl groups of LigW, as the interaction of additional hydroxyl groups with isocyanate disrupts the balance of the foaming mixture [34]. In addition, the LigW/LG 1:1 particles affected the gel and tack-free times less dramatically than the LigW/LG 1:2 particles. For example, the LigW/LG 1:1 particles increased the gel and tack-free times from 80 s to 98 s and from 162 s to 172 s, respectively, while the LigW/LG 1:2 particles showed greater compatibility with the mixture and reduced the gel and tack-free times from 82 s to 76 s and from 161 s to 150 s, thus showing the improved interaction between the foaming mixture and LigW/LG 1:2 particles.

### 3.3. Thermal Conductivity and Microstructure of BioPUR Foams

**Figure 3:**
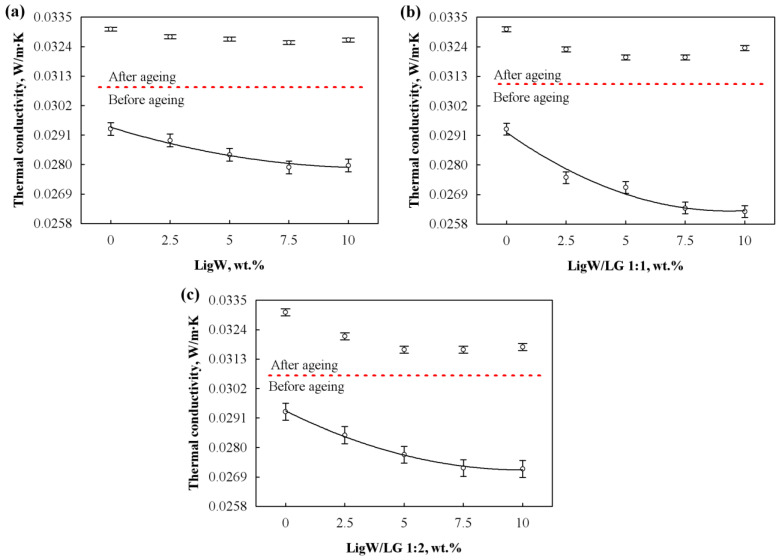
Thermal conductivity before and after ageing of bioPUR foams with: (**a**) LigW particles; (**b**) LigW/LG 1:1 particles; (**c**) LigW/LG 1:2 particles.

Based on the results presented in Figure 3, it can be observed that thermal conductivity reduced with the addition of LigW, LigW/LG 1:1, and LigW/LG 1:2 particles. For instance, the average thermal conductivity value for bioPUR foams with LigW improved by 3.8%, while for foams with LigW/LG 1:1 it improved by 10% and for foams with LigW/LG 1:2 it improved by 7% at 10 wt.% particles compared to the control bioPUR foam. The reduction observed is in great agreement with the closed-cell content results indicated in Table 3. The volumetric percentage of closed cells increased with the addition of particles, regardless of the amount of LG used for modification. However, Barczewski et al. [35] showed that the incorporation of expanded vermiculite resulted in a different tendency. The thermal conductivity was higher for composite foams with the highest closed-cell content, while Członka et al. [36] indicated that walnut shell filler improved the thermal conductivity of composite foams with the highest content of closed cells. The differences between similar studies may be explained by the nature of the filler, its size, surface preparation (if any), proper distribution in the polymeric system, and its ability to interact with the polymer matrix.

In order to quantitatively evaluate the impact of the LigW, LigW/LG 1:1, and LigW/LG 1:2 particles on the thermal conductivity of bioPUR foams, a mathematical–statistical analysis was implemented. It shows that the thermal conductivity for bioPUR foams modified with 0–10 wt.% particles might be approximated by regression Equations (2)–(4):

(2) $$\lambda_{LigW}=0.0294-0.270\cdot 10^{-4}\cdot m_{LigW}+0.122\cdot 10^{-6}\cdot m_{LigW}^{2}$$

(3) $$\lambda_{LigW/LG1:1}=0.0292-0.630\cdot 10^{-3}\cdot m_{LigW/LG1:1}+0.335\cdot 10^{-4}m_{LigW/LG1:1}^{2}$$

(4) $$\lambda_{LigW/LG1:2}=0.0294-0.440\cdot 10^{-3}\cdot m_{LigW/LG1:2}+0.217\cdot 10^{-4}\cdot m_{LigW/LG1:2}^{2}$$

where $\lambda_{LigW}$, $\lambda_{LigW/LG1:1}$ and $\lambda_{LigW/LG1:2}$ are the thermal conductivities of the LigW, LigW/LG 1:1, and LigW/LG 1:2 modified bioPUR foams, respectively, W/(m·K); $m_{LigW}$, $m_{LigW/LG1:1}$, and $m_{LigW/LG1:2}$ are the amounts of LigW, LigW/LG 1:1, and LigW/LG 1:2 particles, respectively, wt.%.

The standard deviations of these dependences are $S_{LigW}=0.000197$ W/(m·K), $S_{LigW/LG1:1}=0.000263$ W/(m·K), and $S_{LigW/LG1:2}=0.000231$ W/(m·K), respectively, while the correlation square ratios—$\eta_{LigW}^{2}=0.905$, $\eta_{LigW/LG1:1}^{2}=0.955$, and $\eta_{LigW/LG1:2}^{2}=0.939$, respectively. These parameters show that the thermal conductivity of the bioPUR foams is 90.5%, 95.5%, and 93.9% dependent, respectively, on the amounts of LigW, LigW/LG 1:1, and LigW/LG 1:2 particles. The dependences are valid for the amounts of particles in the range of 0–10 wt.%.

Furthermore, it can be seen from Figure 3 that thermal conductivity after ageing bioPUR foam samples with unmodified and LG-modified LigW particles increased compared to initial thermal conductivity values. This behaviour of bioPUR foams is attributed to the rapid escape of carbon dioxide at ageing temperature. Comparing the effect of unmodified and LG-modified LigW particles on the aged thermal conductivity value of bioPUR foams, some interesting changes can be seen.

For instance, 7.5 wt.% LigW, LigW/LG 1:1, and LigW/LG 1:2 particles reduce thermal conductivity after ageing by 1.5%, 3.2%, and 4.2%, respectively, compared to control bioPUR foams, while further addition of the particles increases the value of thermal conductivity after ageing, assumingly due to excess of particle loading.

**Figure 4:**
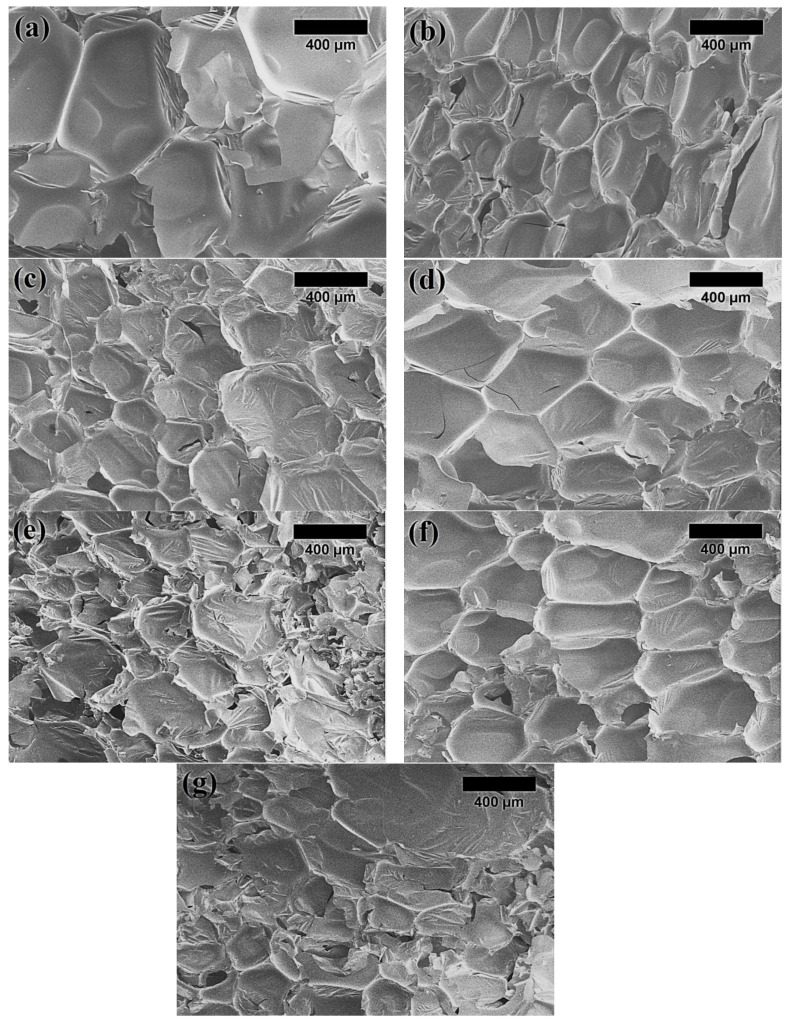
Microstructure of bioPUR foams (magnification ×100): (**a**) control bioPUR; (**b**) bioPUR with 5 wt.% LigW; (**c**) bioPUR with 10 wt.% LigW; (**d**) bioPUR with 5 wt.% LigW/LG 1:1; (**e**) bioPUR with 10 wt.% LigW/LG 1:1; (**f**) bioPUR with 5 wt.% LigW/LG 1:2; (**g**) bioPUR with 10 wt.% LigW/LG 1:2.

An interesting behaviour can be pointed out by observing the apparent density data, reported in Table 4. The effect of the LigW particles would mainly drive the apparent density to decrease with respect to the control bioPUR foam. The average values of apparent density reduced by 9.5–21.5% depending on the amount of particles added. Although the incorporation of LigW/LG 1:1 and LigW/LG 1:2 particles into bioPUR foams resulted in an increase of apparent density, i.e., up to 9.5% for both types of particles, the cause for such changes was the hydroxyl groups in the lignin. Hydroxyl groups are the most reactive functional groups in lignin, and consequently react promptly with isocyanates to form polyurethanes due to the nucleophilic attack of the oxygen from the hydroxyl groups on the electrophilic carbon of the isocyanate groups [37], thus causing the additional expansion and reduction in the apparent density of bioPUR foams with LigW particles, while LG-coated LigW particles prohibit the access of isocyanate to these hydroxyl groups and assure the stability of the bioPUR foams. Interestingly, the average cell size of the bioPUR foams reduced with the addition of LigW particles, as well as LigW/LG 1:1 and LigW/LG 1:2. Although the free hydroxyl groups lead to additional expansion, the LigW particles themselves also work as nucleation centres [38], allowing the formation of smaller cells with thinner walls, as indicated in Table 4 and Figure 4.

It can be seen from Figure 4 that both the cell shape and the homogeneous integrity of the bioPUR foams were changed after the addition of LigW or LG-modified LigW particles. According to the data presented in Table 4, LigW particles reduced the average cell size by a maximum of 62%, LigW/LG 1:1 by 70%, and LigW/LG 1:2 by 60% compared to the control bioPUR foams. Moreover, Figure 4 indicates that up to 5 wt.% particles induce the formation of fine microstructure with regular cells, while the incorporation of 10 wt.% particles stimulates the shaping of the inhomogeneous structure of bioPUR foams regardless of the particle type. This was also in agreement with the studies by Bonab et al. [39] and Bartczak et al. [40], who reported that the lower contents of fillers in polyurethane foams led to a more uniform and homogeneous cell structure.

### 3.4. Dimensional Stability of BioPUR Foams

**Figure 5:**
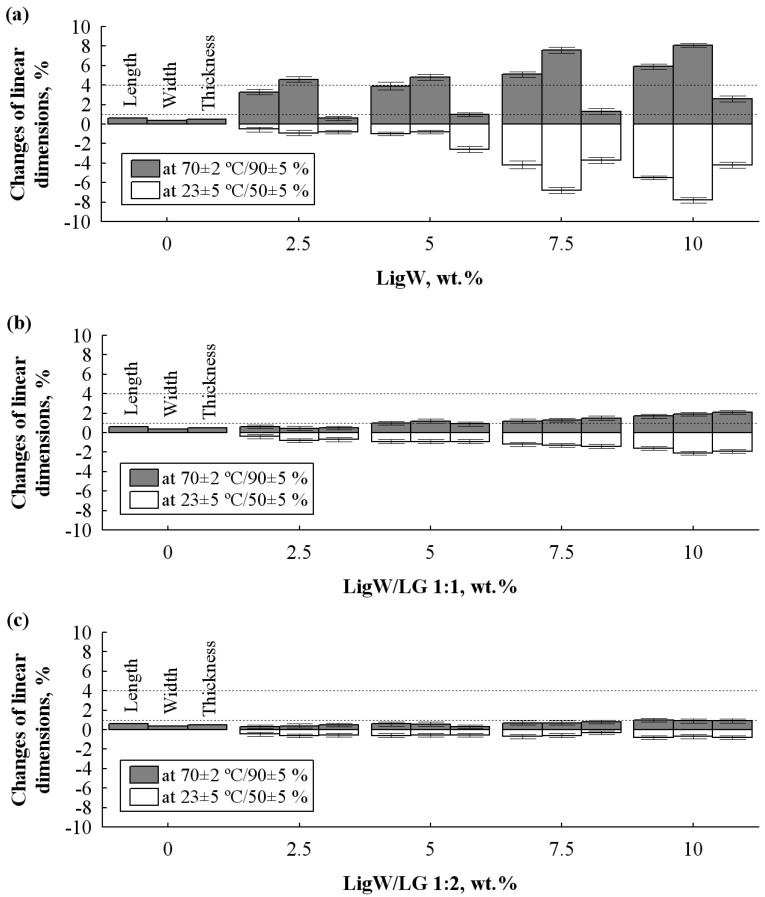
Dimensional stability of bioPUR foams at normal and elevated conditions: (**a**) with LigW particles; (**b**) with LigW/LG 1:1 particles; (**c**) with LigW/LG 1:2 particles. (-----)—the strictest requirements of length, width (≤4%) and thickness (≤1%) at elevated temperature and humidity conditions according to EN 14315-1 [17].

To date, there have been few studies analysing the impact of fillers on the dimensional stability of bioPUR foams [41,42,43] which were synthesised using the system of lower- and higher-functionality polyols. Although the changes in linear dimensions under ambient temperature are not favourable, the sufficient operation in higher temperature and humidity conditions must be validated as well. Therefore, the results of dimensional stability at (23 ± 5) °C/(50 ± 5)% and (70 ± 2) °C/(90 ± 5)% are presented in Figure 5. Obviously, the increase in the amount of LigW particles causes higher dimensional changes of bioPUR foams at ambient and elevated conditions. LigW particles promote additional expansion of the foaming mixtures, thus thinning the cell walls, which are not capable of withstanding the pressure of CO_2_ after the bioPUR foam hardens, as well as the escape of residual CO_2_ during the dimensional stability test at elevated temperature and humidity conditions, which then results in initial shrinkage at ambient temperature and further expansion at increased temperature (Figure 5a). It was established in a previous study [44] that the temperature and diffusion rate are proportional parameters, and it can be pointed out that the temperature enhances the rate of CO_2_ gases penetrating through cell walls because of the energy required for their permeation from the cellular structure to the surrounding environment.

Figure 5b,c shows that the modification of LigW particles with LG at ratios of 1:1 and 1:2 allows dimensionally stable foams to be obtained even with the 10 wt.% LigW/LG 1:1 and LigW/LG 1:2 particles.

Compared to the control bioPUR foam, slight shrinkage, for instance from 0% to a maximum of 2%, for 10 wt.% LigW/LG 1:1 at ambient temperature and expansion, from a maximum of 0.8% to 3% at elevated temperature, for bioPUR foams with LigW/LG 1:1 particles occur due to the increased closed-cell content. Similar observations were made for bioPUR foams modified with LigW/LG 1:2 particles. The most likely reason for the stability of bioPUR foams modified with LigW/LG 1:1 and LigW/LG 1:2 compared to LigW particles might be the fact that LG effectively coats the surface of the particles, thus eliminating the additional expansion factor. Additionally, the higher the ratio of LG used, the higher the efficacy that is observed. All bioPUR foams modified with LigW/LG 1:1 and LigW/LG 1:2 particles conformed with the requirements of the product standard and can be successfully used for thermal insulation of building envelopes.

### 3.5. Mechanical Properties of BioPUR Foams

**Figure 6:**
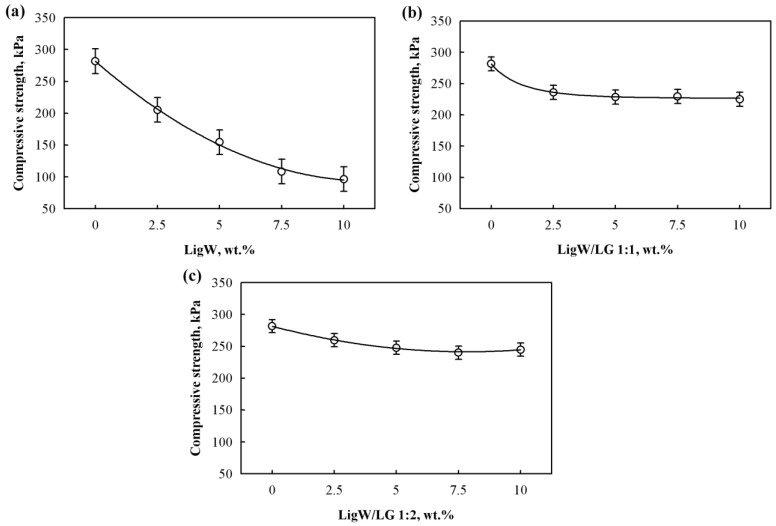
Compressive strength of bioPUR foams with: (**a**) LigW particles; (**b**) LigW/LG 1:1 particles; (**c**) LigW/LG 1:2 particles.

The mechanical properties mostly depend on the cell structure of the foams, the uniformity and size of the cells, and also the apparent density. A more compact cellular structure determines a higher apparent density; consequently, more material per unit area decides the higher mechanical strength of the final product [45]. Figure 6 shows the compressive strength values of bioPUR foams modified with LigW, LigW/LG 1:1, and LigW/LG 1:2 particles. An increase in the amount of each particle type in the bioPUR foams reduced the compressive strength values.

Figure 6a shows that, compared to the control bioPUR foams, bioPUR foams modified with LigW particles had a compressive strength reduced by a maximum of 65%. This can be attributed to the fact that, with a higher amount of LigW particles, the apparent density decreased, and the lower apparent density resulted in reduced compressive strength of the bioPUR foams. Additionally, this is also due to the lower cross-link density of such foams. Even though the addition of LigW/LG 1:1 and LigW/LG 1:2 particles (Figure 5b,c) reduced the compressive strength of the modified bioPUR foams, it was much higher compared to bioPUR foams with LigW particles. For example, 2.5 wt.% LigW/LG 1:1 and LigW/LG 1:2 particles increased the parameter by 15% and 32%, respectively; 5 wt.% LigW/LG 1:1 and LigW/LG 1:2 by 48% and 57%, respectively; 7 wt.% LigW/LG 1:1 and LigW/LG 1:2 by 112% and 121%, respectively; and 10 wt.% LigW/LG 1:1 and LigW/LG 1:2 by 133% and 153%, respectively. The increase in strength of the bioPUR foams modified with LigW/LG 1:1 and LigW/LG 1:2 compared to bioPUR foams modified with LigW particles is related to the formation of cross-links between compatible fillers and the bioPUR matrix. This mechanism was studied and reported in a few studies as being able to induce a gain in some mechanical properties [46,47,48], although such filler–matrix interactions may not be strong enough to impart increases in mechanical performance and overcome the strength of control bioPUR foams, as the filler particles attach themselves to the cell walls, encapsulating blowing gas, and turn into nucleation sites, which weaken, and even damage, the edges of cells due to the weaker interaction between hydroxyl groups on the surface of the particles and isocyanate groups.

It was also observed that the compressive strength results change in a certain manner. Therefore, in order to quantitatively evaluate the impact of the LigW, LigW/LG 1:1, and LigW/LG 1:2 particles on the compressive strength of bioPUR foams, mathematical–statistical analysis was also implemented. It shows that the compressive strength for bioPUR foams modified with 0–10 wt.% particles might be approximated by regression Equations (5)–(7):

(5) $$\sigma_{LigW}=281.8-33.96\cdot m_{LigW}+1.528\cdot m_{LigW}^{2}$$

(6) $$\sigma_{LigW/LG1:1}=33.10\cdot (1-(-7.51)\cdot \exp(-0.178\cdot m_{LigW/LG1:1}^{0.159}))$$

(7) $$\sigma_{LigW/LG1:2}=281.7-10.30\cdot m_{LigW/LG1:2}+0.657\cdot m_{LigW/LG1:2}^{2}$$

where $\sigma_{LigW}$, $\sigma_{LigW/LG1:1}$, and $\sigma_{LigW/LG1:2}$ are the compressive strengths of the LigW, LigW/LG 1:1, and LigW/LG 1:2 modified bioPUR foams, respectively, W/(m·K); $m_{LigW}$, $m_{LigW/LG1:1}$ and $m_{LigW/LG1:2}$ are the amounts of LigW, LigW/LG 1:1, and LigW/LG 1:2 particles, respectively, wt.%. The standard deviations of these dependences are $S_{LigW}=14.1$ kPa, $S_{LigW/LG1:1}=8.20$ kPa, and $S_{LigW/LG1:2}=7.31$ kPa, respectively, while the correlation square ratios are $\eta_{LigW}^{2}=0.967$, $\eta_{LigW/LG1:1}^{2}=0.899$, and $\eta_{LigW/LG1:2}^{2}=0.836$, respectively. These parameters show that the compressive strength of bioPUR foams is 96.7%, 89.9%, and 83.6% dependent, respectively, on the amounts of LigW, LigW/LG 1:1, and LigW/LG 1:2 particles. The dependences are valid for the amounts of particles in the range of 0–10 wt.%.

**Figure 7:**
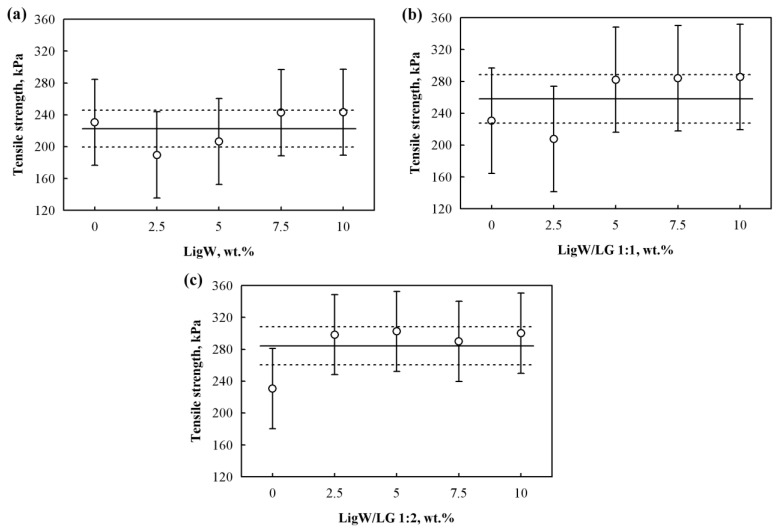
Tensile strength of bioPUR foams with: (**a**) LigW particles; (**b**) LigW/LG 1:1 particles; (**c**) LigW/LG 1:2 particles.

Figure 7 presents the tensile strength results for bioPUR foams prepared with unmodified and LG-modified LigW particles. It can be seen that all the bioPUR foams obtained without taking into account the hydroxyl values of LigW (Figure 7a) had tensile strength values ranging from 190 kPa to 245 kPa. The maximum value of tensile strength was obtained with the 10 wt.% LigW particles and, compared to the control bioPUR foam, the increase was insignificant. This could be attributed to the hydroxyl groups on the LigW particles and their reaction with isocyanate. According to Olszewski et al. [49], this reaction reduces the number of isocyanate groups that are necessary for polymerization reaction, thus weakening the polymer chains in the bioPUR foams.

According to the data presented in Figure 7b,c, bioPUR foams with LigW/LG 1:1 had tensile strength values ranging from 208 kPa to 285 kPa and with LigW/LG 1:2, from 298 kPa to 300 kPa. The effectiveness of LigW/Lg 1:1 particles is visible from 5 wt.% (the maximum increase is observed to be 24% compared to the control bioPUR foam), while for bioPUR foams with LigW/LG 1:2, the efficacy was obtained from 2.5 wt.% (the maximum increase is observed to be 30% compared to the control bioPUR foam), indicating that a higher amount of LG better “locks” the hydroxyl groups on the surface of the LigW particles and allows higher mechanical performance of bioPUR foams to be achieved. Generally speaking, for the LigW, LigW/LG 1:1 and LigW/LG 1:2 particles, the differences in their shape, size, surface modification, and amount change their dispersion and interfacial bonding in the polymer matrix, consequently affecting the mechanical properties of the bioPUR foams [50].

### 3.6. Water and Water Vapour Resistance Properties of BioPUR Foams

**Figure 8:**
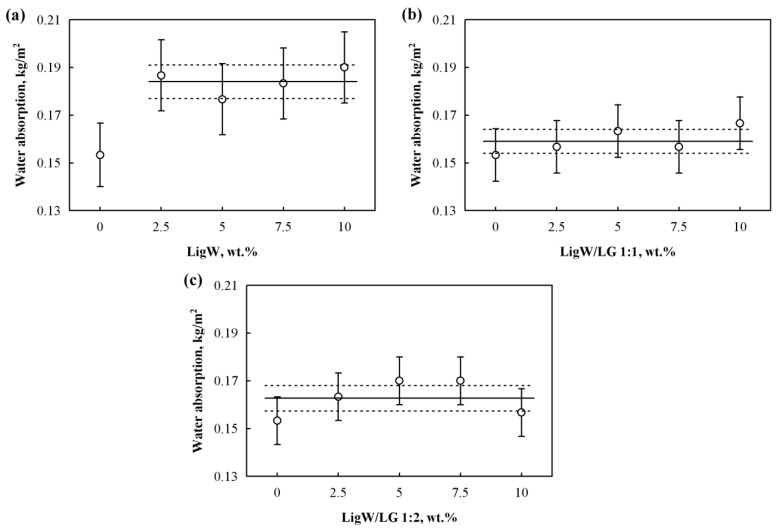
Short-term water absorption of bioPUR foams with: (**a**) LigW particles; (**b**) LigW/LG 1:1 particles; (**c**) LigW/LG 1:2 particles.

Water absorption properties were determined on the bioPUR foams developed with different types of LigW particles (Figure 8). The water absorption values were found to be constant rather than increasing with the addition of unmodified LigW particles for the bioPUR foams. Even though the bioPUR foams with LigW particles had a higher closed-cell content compared to the control bioPUR foam, Figure 8a shows that the values of water absorption increased by a maximum of 27% for bioPUR foam with 10 wt.% LigW particles. Very similar values were obtained for the remaining amounts of LigW particles, and the minimal changes in average values are insignificant. The reason for such results is that bioPUR foams with unmodified LigW particles contain more unreacted polyol and hydroxyl groups because of the reaction between lignin and NCO, causing a high hygroscopicity of the bioPUR foam matrix. A similar water absorption trend was reported for polyurethane foams with brown-rotted spruce filler [51]. Santos et al. [52] also stated that lignin as a filler reduces the hydrophobicity of the foams due to the polar groups in the structure.

Figure 8b,c show that LG-coated LigW particles result in bioPUR foams with quite stable water absorption. Apparently, the LG increased the hydrophobicity of the LigW particles and, compared to bioPUR foams with LigW particles, the water absorption was significantly reduced and close to that of the control bioPUR foam. Similar trends can also be seen for the results of the water vapour diffusion resistance factor in Table 5. A higher amount of LigW particles increases the foam’s ability to transmit more water vapour, while LG-modified LigW particles only slightly improve the water vapour diffusion resistance factor.

For instance, 10 wt.% LigW/LG 1:1 particles increased the parameter by a maximum of 9%, while the same amount of LigW/LG 1:2 particles increased the parameter by a maximum of almost 12% compared to the control bioPUR foam.

The slight improvement in the water vapour diffusion resistance factor may be attributed to the fact that the bioPUR foams modified with LigW/LG 1:1 and LigW/LG 1:2 particles had a higher content of closed cells, and to the assumption that LG-coated particles might act as a barrier for water and water vapour penetration. As previously determined [44], some particles and polymeric matrix can have sufficient interfacial adhesion to prevent the formation of cracks and cavities, inducing the smooth penetration of water molecules during water absorption testing and water vapour during water vapour permeability testing.

## 4. Conclusions

BioPUR foam was modified with 2.5–10 wt.% LigW and LG-modified LigW particles. The impact of the LigW and modified LigW particles at different LG ratios on selected properties of the bioPUR foams, such as rheology, apparent density, thermal conductivity, dimensional stability at ambient and elevated conditions, compressive and tensile strengths, short-term water absorption, and water vapour permeability, was investigated. It was determined that LigW particles modified with LG at a ratio of 1:2 led to the greatest improvement in the performance of the bioPUR foams. The dynamic viscosity of polyol premixed with 10 wt.% LigW/LG 1:2 was reduced by 70% while the foaming times were significantly improved compared to bioPUR foams with unmodified LigW particles. The incorporation of LigW/LG 1:2 particles allowed fully dimensionally stable bioPUR foams to be obtained with linear changes as low as 1%. Additionally, 10 wt.% LigW/LG 1:1 particles improved thermal conductivity by 6.7%, while 10 wt.% LigW/LG 1:2 improved thermal conductivity by 3.2% compared to bioPUR foams with unmodified LigW particles. Compressive and tensile strengths that were higher by 153% and 23.5, respectively, were also achieved for the highest amount of LigW/LG 1:2 particles incorporated. Water absorption and water vapour permeability tests indicated that the addition of 10 wt.% LigW/LG 1:2 particles does not degrade the moisture properties and is suitable for application in thermal insulating bioPUR foams.

## Figures and Tables

**Table 1:** Composition of BioPUR foams.

Materials, Pbw	BioPUR	BioPUR with LigW	BioPUR with LigW/LG 1:1	BioPUR with LigW/LG 1:2
BioPolyol RD	60	60	60	60
Petol PZ 400-4G	40	40	40	40
Distilled water	2.5	2.5	2.5	2.5
Polycat 9	1	1	1	1
ST-52	3	3	3	3
LigW, wt.%	0	2.5; 5; 7.5; 10	0	0
LigW/LG 1:1, wt.%	0	0	2.5; 5; 7.5; 10	0
LigW/LG 1:2, wt.%	0	0	0	2.5; 5; 7.5; 10
Isocyanate index	125			

**Table 2:** Elemental composition of LigW and LigW/LG.

Type of Particles	The Main Elements, %							
	C	O	Na	Al	Si	S	Ca	Fe
LigW	64.17	34.96	0.04	0.16	0.18	0.09	0.19	0.13
LigW/LG 1:1	48.48	38.12	4.87	0.10	7.35	0.39	0.31	0.27
LigW/LG 1:2	45.15	39.36	6.04	0.09	8.56	0.31	0.23	0.23

Note: The missing small percentages are of K and Mg.

**Table 3:** Foaming parameters and characteristics of polyol mixture.

Amount of Lignin Waste, wt.%	Characteristic				
	Dynamic Viscosity, mPa s	Cream Time, s	Gel Time, s	Tack-Free Time, s	Peak Temperature *, °C
bioPUR mixture with unmodified LigW					
0	199 ± 10	26 ± 2	74 ± 2	145 ± 2	134 ± 3
2.5	286 ± 12	27 ± 1	80 ± 2	167 ± 3	138 ± 2
5	409 ± 15	28 ± 2	89 ± 2	176 ± 2	142 ± 3
7.5	649 ± 12	29 ± 1	91 ± 2	179 ± 2	146 ± 4
10	1584 ± 9	29 ± 3	96 ± 2	198 ± 3	149 ± 2
bioPUR mixture with LigW/LG ratio 1:1					
2.5	246 ± 20	28 ± 2	80 ± 3	162 ± 3	132 ± 2
5	319 ± 19	28 ± 2	89 ± 2	173 ± 2	130 ± 2
7.5	391 ± 14	26 ± 2	93 ± 2	174 ± 2	125 ± 3
10	486 ± 18	25 ± 1	98 ± 3	172 ± 2	121 ± 2
bioPUR mixture with LiqW/LG ratio 1:2					
2.5	256 ± 15	30 ± 3	82 ± 2	161 ± 1	125 ± 4
5	375 ± 16	28 ± 2	79 ± 1	169 ± 2	121 ± 3
7.5	407 ± 13	28 ± 2	81 ± 3	165 ± 2	116 ± 2
10	491 ± 10	18 ± 2	76 ± 1	150 ± 1	109 ± 2

* Peak temperature is the highest temperature reached during the foaming process.

**Table 4:** Structural analysis results of bioPUR foams.

Amount of Lignin Waste, wt.%	Characteristic		
	Apparent Density, kg/m^3^	Closed-Cell Content, vol.%	Cell Size, μm
bioPUR with unmodified LigW			
0	42 ± 2	84 ± 2	642 ± 12
2.5	38 ± 3	85 ± 2	484 ± 14
5	36 ± 2	91 ± 2	336 ± 21
7.5	35 ± 2	92 ± 3	298 ± 18
10	33 ± 2	91 ± 2	242 ± 15
bioPUR with LigW/LG ratio 1:1			
2.5	42 ± 3	92 ± 2	508 ± 10
5	42 ± 3	92 ± 3	412 ± 13
7.5	45 ± 2	96 ± 2	320 ± 19
10	46 ± 2	96 ± 3	192 ± 21
bioPUR with LiqW/LG ratio 1:2			
2.5	43 ± 4	91 ± 2	421 ± 14
5	44 ± 2	93 ± 2	304 ± 16
7.5	46 ± 3	95 ± 2	288 ± 14
10	46 ± 2	96 ± 2	257 ± 18

**Table 5:** Water vapour permeability of bioPUR foams.

Amount of Filler, wt.%	Water Vapour Diffusion Resistance Factor μ, n. d.			
	bioPUR	bioPUR with LigW	bioPUR with LigW/LG 1:1	bioPUR with LigW/LG 1:2
0	43 ± 2			
2.5		38 ± 4	45 ± 4	46 ± 3
5		37 ± 2	46 ± 3	47 ± 3
7.5		35 ± 4	46 ± 2	48 ± 4
10		34 ± 3	47 ± 4	48 ± 3

Note: μ has no dimensions (n. d.).

## Data Availability

Not applicable.
